# Disrupted metabolic connectivity in dopaminergic and cholinergic networks at different stages of dementia from ^18^F-FDG PET brain persistent homology network

**DOI:** 10.1038/s41598-021-84722-8

**Published:** 2021-03-08

**Authors:** Tun-Wei Hsu, Jong-Ling Fuh, Da-Wei Wang, Li-Fen Chen, Chia-Jung Chang, Wen-Sheng Huang, Hsiu-Mei Wu, Wan-Yuo Guo

**Affiliations:** 1 Department of Radiology, Taipei Veterans General Hospital, No. 201, Sec. 2, Shipai Rd., Beitou District, Taipei, 11217 Taiwan; 2 Faculty of Medicine, School of Medicine, National Yang-Ming University, Taipei, Taiwan; 3 Division of General Neurology, Neurological Institute, Taipei Veterans General Hospital, No. 201, Sec. 2, Shipai Rd., Beitou District, Taipei, 11217 Taiwan; 4 Brain Research Center, National Yang-Ming University, Taipei, Taiwan; 5 Institute of Information Science, Academia Sinica, Taipei, Taiwan; 6 Institute of Brain Science, School of Medicine, National Yang-Ming University, Taipei, Taiwan; 7 Department of Nuclear Medicine, Taipei Veterans General Hospital, Taipei, Taiwan; 8 Integrated PET/MR Imaging Center, Taipei Veterans General Hospital, Taipei, Taiwan

**Keywords:** Molecular medicine, Neurology, Diseases, Metabolic disorders, Positron-emission tomography, Developmental biology, Ageing, Imaging, Metabolomics, Neuroscience, Cognitive ageing, Cognitive neuroscience, Diseases of the nervous system, Neural ageing

## Abstract

Dementia is related to the cellular accumulation of β-amyloid plaques, tau aggregates, or α-synuclein aggregates, or to neurotransmitter deficiencies in the dopaminergic and cholinergic pathways. Cellular and neurochemical changes are both involved in dementia pathology. However, the role of dopaminergic and cholinergic networks in metabolic connectivity at different stages of dementia remains unclear. The altered network organisation of the human brain characteristic of many neuropsychiatric and neurodegenerative disorders can be detected using persistent homology network (PHN) analysis and algebraic topology. We used ^18^F-fluorodeoxyglucose positron emission tomography (^18^F-FDG PET) imaging data to construct dopaminergic and cholinergic metabolism networks, and used PHN analysis to track the evolution of these networks in patients with different stages of dementia. The sums of the network distances revealed significant differences between the network connectivity evident in the Alzheimer’s disease and mild cognitive impairment cohorts. A larger distance between brain regions can indicate poorer efficiency in the integration of information. PHN analysis revealed the structural properties of and changes in the dopaminergic and cholinergic metabolism networks in patients with different stages of dementia at a range of thresholds. This method was thus able to identify dysregulation of dopaminergic and cholinergic networks in the pathology of dementia.

## Introduction

Dementia is a neurodegenerative disease characterised by a progressive and chronic loss of function in cognitive, motor, sensory, and other brain systems^1^. The causes of Alzheimer’s disease (AD) and some other types of dementia are related to cellular pathologies, such as β-amyloid plaques^2^, tau aggregates^3^ and α-synuclein aggregates^4^ or to neurotransmitter deficiencies in the dopaminergic^5^ and cholinergic^6^ systems. Alterations in cholinergic circuity have been reported in disorders of attention and cognition^7,8^. Abnormal it contributes to memory and reward dysfunction in AD^9^. Dopamine appears to be particularly involved in the regulation of cognitive processes, and also has a functional relationship with the cholinergic system^10^. Furthermore, the accumulation of α-synuclein affects cholinergic and dopaminergic deficits.

Brain ^18^F-fluorodeoxyglucose positron emission tomography (^18^F-FDG PET) has been used for many years to diagnose dementia. The main advantage of ^18^F-FDG PET is its high sensitivity in detecting pathologies at the molecular level, due to the transportation of ^18^F-fluorodeoxyglucose into the intracellular space by glucose-1 transporters and its subsequent phosphorylation through the hexokinase reaction. ^18^F-FDG PET is used to measure energy consumption in neurons^11^, and it is associated with synaptic density and function. This method is sensitive to pathological changes in metabolic and biochemical processes, in contrast to magnetic resonance imaging, which can only visualise morphological changes. Using the ^18^F-FDG PET metabolic network, local neural activity, disconnection, and neuropathology effects can be observed. Each brain region can be assumed to exchange information directly or indirectly with other parts of the network through synchronized fluctuations in glucose uptake. Thus, we converted the brain images we obtained using ^18^F-FDG PET into a metabolic network and analysed the dopaminergic^5^ and cholinergic^6^ network connectivity between brain regions.

Network science involves measuring the similarity or difference between networks based on topology. Graph theory can be used to investigate the attributes of brain function and structure, and to do this the brain must be completely characterised as a network to understand brain function^12^. However, classical graph theory measures are usually based on either node or edge measurements, without considering aspects of network topology, such as connected components. Graph theory measures, such as the characteristic path length (CPL)^13^, network diameter (ND)^14^, and modularity (Mod)^15,16^, have been widely used to study the metabolic brain networks of patients with AD and mild cognitive impairment (MCI). These studies have reported topological alterations, resulting in lower efficiency, based on CPL, ND and disrupted Mod organisation^17^. The persistent homology network (PHN)^18^ is a mathematical tool in computational topology used for analysing the topological features of data that are persistent across in-network. PHN analysis was proposed as a precise and robust method for detecting differences in brain connectivity in AD^19^. It uses a topological feature (such as the number of connected brain regions)^20^ and a connected aggregation cost. This PHN feature is a monotonically decreasing convergence function and, when plotted across all possible filtration values, enables tracking network evolution between different groups. The connected component aggregation cost is derived from the minimum spanning tree of the network; thus, it can be considered the least efficient glucose metabolism pathway^21^ required for the evolution of the fully connected components.

The role of dopaminergic and cholinergic network connectivity in different stages of dementia is still unclear. PHN analysis can indicate how subcomponents of circuits are integrated into full connection when the filtration value increases. We studied changes in brain connectivity at different stages of dementia through the topological patterns of the dopaminergic and cholinergic networks derived from PHN analysis and successfully distinguished alterations in metabolic connectivity in the brain using ^18^F-FDG PET imaging. In this way we were able to identify dysregulation of dopaminergic and cholinergic networks in the pathology of dementia.

## Results

We classified the values of the graph-based network indices CPL, ND, and eigenvector centrality (EC) into four groups based on their weighted networks after edge filtering; their corresponding *P* values passed the statistical threshold (10,000 permutations; *P* < 0.001). We also obtained the multinetwork according to graph filtration (λ) and computed four separate slopes of the integrated persistent plot of the area under the curve (SIP AUC) for patients with AD, MCI, and subjective cognitive decline (SCD), as well as for healthy controls (HC). All brain network parameters are presented in Supplementary Table S1.

### Dopaminergic network analysis

**Figure 1:**
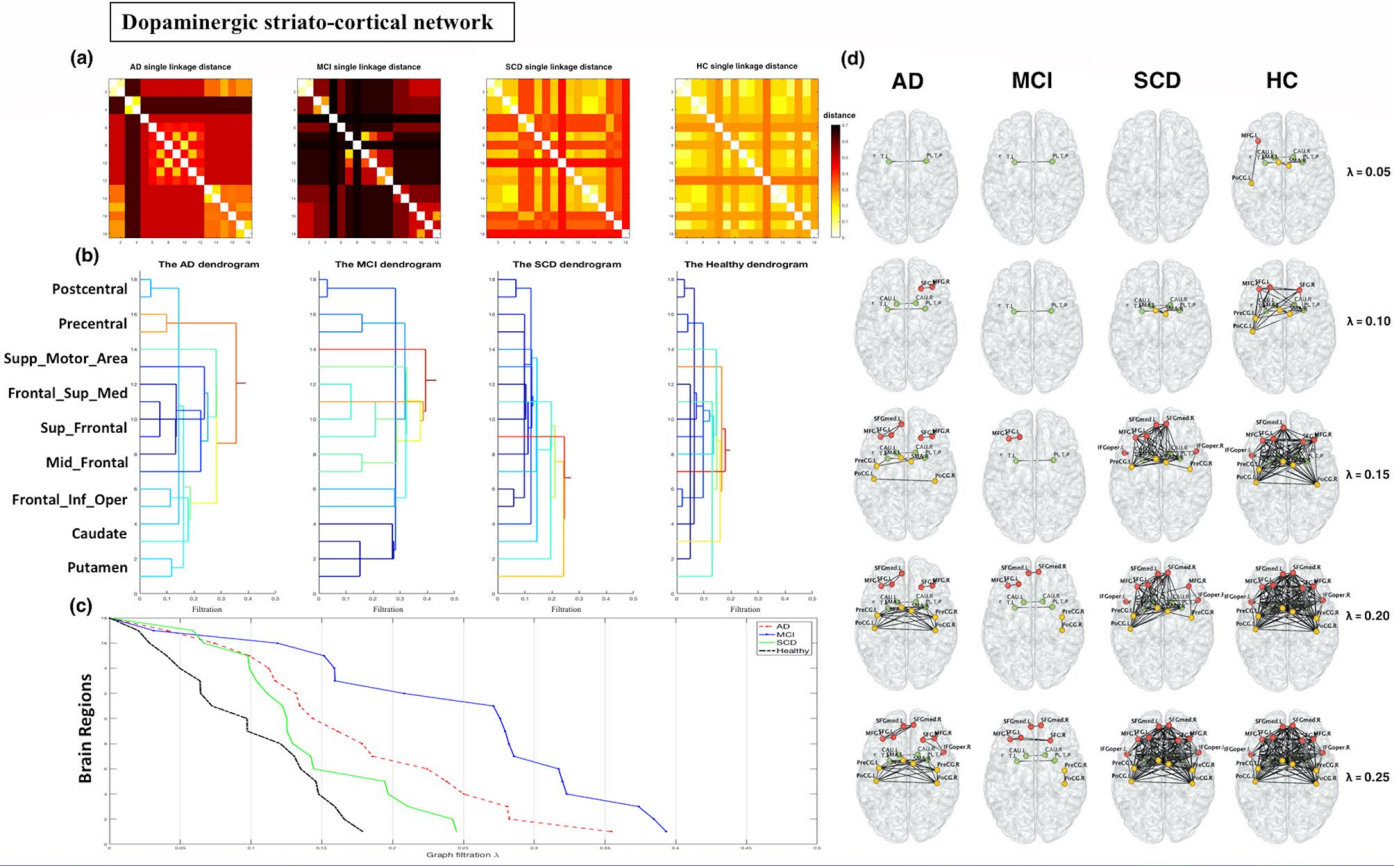
Dopaminergic network analysis. The dorsal dopamine pathway was affected in the Alzheimer’s disease (AD) and mild cognitive impairment (MCI) groups, showing a delay in connectivity and changes in configuration. (**a**) Single linkage distance (SLD) in the AD, MCI, subjective cognitive decline (SCD), and healthy control (HC) groups, obtained from the original correlation-based distance. (**b**) Single linkage dendrograms for the AD, MCI, SCD, and HC groups. The vertical and horizontal axes represent brain regions and graph filtration. The connected network is divided into smaller regions when the distance (graph filtration) decreases. (**c**) The corresponding persistent features of each group at different scales of graph filtration and (**d**) striatocortical network maps constructed using five different filtration values: 0.05, 0.10, 0.15, 0.20, and 0.25.

For the striatocortical pathways of the dopaminergic network, the single linkage distance (SLD) and dendrogram for each group are displayed in Fig. 1a,b. The SLD represents the functional distance between two brain regions. When λ increased, the dendrogram indicated that modularity occurred from the dorsal striatum to the prefrontal and sensorimotor cortices and that the supplementary motor regions had large delays in connectivity in patients with AD and MCI, but most markedly for MCI. The models showed a global increase in SLD for each connected region in the AD and MCI groups compared with the SCD and HC groups. To visualise network changes in the striatocortical pathways of the dopaminergic network during graph filtration, representative graphs are displayed at different filtration thresholds (λ = 0.05, 0.10, …, 0.25; Fig. 1c,d). When λ increased, the clustering of brain connections occurred more slowly and at a longer distance (less efficiency in the network) in the AD and MCI groups than in the HC and SCD groups. The pattern of delay was observed as follows: MCI > AD > SCD > HC. Moreover, we observed a significant difference between the CPL and SIP AUC values of various group pairs (Supplementary Table S1).

For the mesolimbic pathways of the dopaminergic network, the SLD and dendrogram for each group are displayed in Supplementary Figure S1a and b. The dendrograms revealed that for higher values of λ, clustering occurred later in the frontal lobe, amygdala, and anterior and posterior cingulate cortex than in other regions in the MCI group. Network changes in the mesolimbic pathways of the dopaminergic network are presented in Supplementary Figure S1c and d. For higher values of λ, the brain connection clustering was slower and less efficient in the AD and MCI groups than in the HC and SCD groups. No significant differences were noted between the SIP AUC values for the four groups, whereas a significant difference was noted between the CPL values for the MCI and SCD groups (Supplementary Table S1).

### Cholinergic network analysis

**Figure 2:**
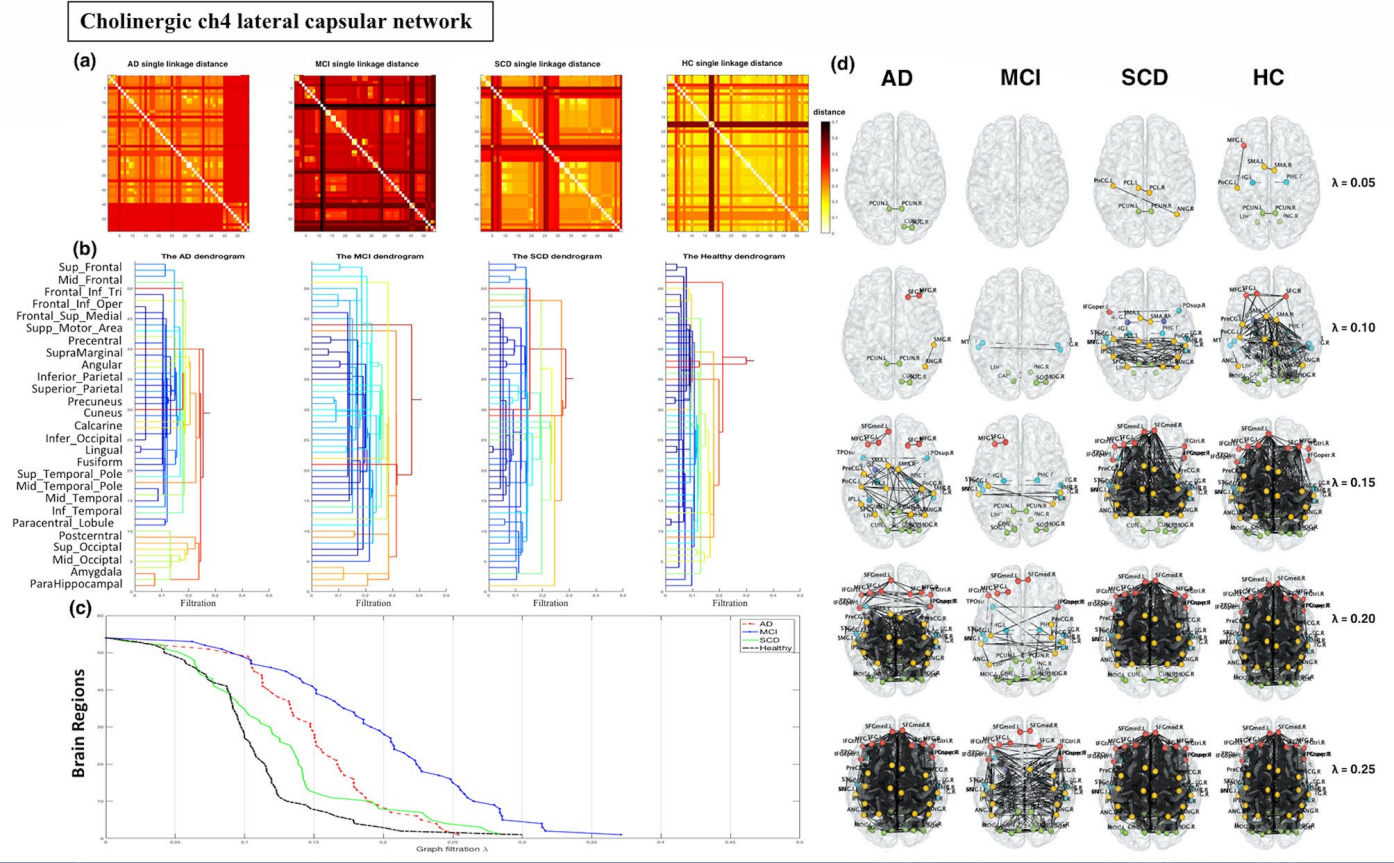
Cholinergic network analysis of the pathways of the Ch4 lateral capsular division reveal a greater distance in the temporal lobe, frontal lobe, and parietal lobe in the Alzheimer’s disease (AD) and mild cognitive impairment (MCI) groups, particularly in the MCI group. (**a**) Single linkage distance (SLD) in the AD, MCI, subjective cognitive decline (SCD), and healthy control (HC) groups, obtained from the original correlation-based distance. (**b**) Single linkage dendrograms for the AD, MCI, SCD, and HC groups. Vertical and horizontal axes represent brain regions and graph filtration, respectively. When the distance (graph filtration) decreases, the connected network is divided into smaller regions. (**c**) The corresponding persistent features of each group at different scales of graph filtration and (**d**) Ch4 lateral capsular projection network maps constructed using five different filtrations values: 0.05, 0.10, 0.15, 0.20, and 0.25.

For the Ch4 lateral capsular pathway of the cholinergic network, the SLD and dendrogram for each group are displayed in Fig. 2a,b. The dendrograms revealed that for higher values of λ, the distance in the temporal, frontal and parietal lobes was greater in the AD and MCI groups, especially the MCI group. In addition, the following delay pattern could be observed: MCI > AD > SCD > HC. To visualise network changes within the Ch4 lateral capsular pathway of the cholinergic network during graph filtration, representative graphs are displayed at various filtration thresholds (λ = 0.05, 0.10, …, 0.25; Fig. 2c,d). For higher values of λ, the brain connection clustering was less efficient and less dense in the AD and MCI groups than in the HC and SCD groups. We observed a significant difference in the SIP AUC for the Ch4 lateral capsular pathway network for the MCI and HC groups and for the MCI and SCD groups (Supplementary Table S1).

For the Ch1–3 pathways of the cholinergic network, the SLD and dendrogram for each group are displayed in Supplementary Figure S2a and b. The dendrograms revealed that clustering occurred later and at a greater distance in the hippocampus and parahippocampus than in other regions in the AD and MCI groups. Network changes in the Ch1–3 pathways of the cholinergic network are displayed at various filtration thresholds in Supplementary Figure S2c and d. For higher values of λ, the brain region clustering was slower and less efficient in the AD and MCI groups than in the HC and SCD groups.

For the Ch4 medial pathway of the cholinergic network, the SLD and dendrogram for each group are displayed in Supplementary Figure S3a and b. The dendrograms revealed that clustering occurred later in the frontal gyrus than in other regions in the AD and MCI groups. The frontal regions were less connected with the cingulate cortex in the AD, MCI, and SCD groups. Network changes in the Ch4 medial pathway of the cholinergic network are displayed at various filtration thresholds in Supplementary Figure S3c and d. For higher values of λ, the brain connection clustering was sparser in the AD, MCI, and SCD groups.

For the Ch4 lateral perisylvian pathway of the cholinergic network, the SLD and dendrogram for each group are displayed in Supplementary Figure S4a and b. The dendrograms revealed a greater distance for the Herschel gyrus, insula and olfactory cortex than for the other regions in the AD and MCI groups. Network changes in the Ch4 lateral perisylvian pathway during graph filtration of the cholinergic network are displayed at various filtration thresholds (λ = 0.05, 0.10, …, 0.25) in Supplementary Figure S4c and d. For higher values of λ, brain connection clustering was slower and less efficient in the AD and MCI groups than in the HC and SCD groups.

In the Ch5–6 pathway of the cholinergic network, the thalamic and midbrain regions were the most affected in the AD and MCI groups. The SLD and dendrogram for each group are displayed in Supplementary Figure S5a and b. The dendrograms revealed that for higher values of λ, the distance in the ventral striatum and brainstem was greater in the AD and MCI groups. To visualise network changes during graph filtration of the Ch5–6 pathway in the cholinergic network, representative graphs are displayed at various filtration thresholds (λ = 0.05, 0.10, …, 0.25) in Supplementary Figure S5c and d. For higher values of λ, the brain connection clustering was less efficient and less dense in the AD and MCI groups than in the HC and SCD groups.

The network parameters of the cholinergic network were a few pairs showed a significant difference (Supplementary Table S1).

## Discussion

In the metabolism network, each brain region exchanges information directly or indirectly with other parts of the network, as indicated by synchronized fluctuations in glucose uptake. We demonstrated that PHN analysis of ^18^F-FDG PET data for patients with different stages of dementia, with different levels of glucose metabolism connectivity, could reveal direct neurotransmitter networks and topological differences. The AD and MCI groups showed neurotransmitter network deficiencies in their dopaminergic and cholinergic networks and had a topological organisation indicating a less efficient topology structure. The PHN analysis revealed whether these brain regions remained intact or had been altered in each group. Importantly, these findings can locate the brain regions that change with disease progression. Ultimately, they may help in assessing the effects of treatments or other interventions on the brain. The hypothesized presence of differences in neurotransmitter networks between patients with MCI or AD and HCs or patients with SCD was confirmed.

The relationship between the dopaminergic network and amyloid-beta pathology is unclear^22^, and their involvement in AD is debatable^23,24^. In general, the release of dopamine transmitter in the caudate, putamen, hippocampus, and frontal cortex of the brain decreases during ageing^25^. The network containing the striatocortical pathways (projections to the sensory–motor cortex) is involved in controlling voluntary movements. The prefrontal cortices are implicated in executive function, and distinct areas of the prefrontal cortex have strong functional connections with striatocortical pathways^26^. The mesolimbic pathway, targeting the hippocampi, cerebral cortex and nucleus accumbens, is responsible for cognitive and behavioural signs. Both the prefrontal cortices and the mesolimbic pathway may be involved in AD progression^5^. The decline of or a deficit in the dopaminergic pathway is predictive of ‘unsuccessful ageing’ and is considered an indicator of frailty in older adults^27^. The CPL and SIP AUC are directly related to the local topology and efficiency of the network. We observed significant differences in the CPL and SIP AUC among the groups. The AD and MCI groups showed slower network integration rates and lower efficiency than the SCD and HC groups in executive and motor functions of the striatocortical pathway network. Some studies have shown strong correlations between dopamine depletion in the striatocortical pathway and deficits in executive function, as indicated using the object alternation task^28^ and the Stroop test^29^. In addition, other studies have indicated that the dopamine and choline systems are tightly interconnected and involved in various cognitive functions, such as memory, attention, and learning^30–32^.

The limbic and paralimbic cortices of the brain receive the heaviest cholinergic input from Ch4 and are also the main sources of mutual projections back to the nucleus basalis. This limbic affiliation explains the role of the basal nucleus in regulating the influence of incoming sensory information and memory. Several studies have reported that AD affects cholinergic system degeneration^33,34^. Cholinergic hypofunction is related to memory deficit progression with ageing. Normal ageing is accompanied by a gradual loss of cholinergic function. Our results showed significant differences in the CPL and SIP AUC of the Ch4 lateral capsular pathway of the cholinergic network among the dementia groups: the AD and MCI groups had slower network integration and lower efficiency in the subbranches of the cholinergic network than the SCD and HC groups. This pathology in Ch4 eventually leads to the degeneration of the part of the cholinergic network, which Ch4 sends to the cerebral cortex. The early involvement of Ch4 has an amplifying effect on Alzheimer’s pathology, because degeneration of Ch4 can perturb neurotransmission in all cortical areas^35^. Our results are consistent with the idea that the cholinergic network has a high demand for energy production and therefore should respond more sensitively to ageing-related energy (glucose) deprivation^36^. According to our PHN analysis results, the MCI group had slower process and sparser connectivity than the AD group; the MCI group may thus have an increased risk of AD (approximately 20% conversion)^37,38^. Cholinergic dysfunction plays a key role in the clinical development of MCI. Previous observations have suggested that disruptions in the metabolic processes of the cholinergic system lead to the full dementia deficits seen in AD, and that this disruption can be an early marker of disease progression^39^. Dysfunction of the cholinergic system can be treated pharmacologically with cholinergic medication, which can ameliorate the cholinergic deficit during the early stages of the disease and retard disease progression.

This study has some strengths and limitations. Traditional graph theory analysis identifies one fixed threshold, which may or may not completely characterise the brain structure network. In contrast, PHN analysis considers topological changes at all possible thresholds, which provides a more complete characterisation of the brain network. In this study, we showed that in a persistent homology context, network evolution can be represented by bar codes. The anatomical regions were based on neurotransmitter mapping and histopathology, however only anatomical regions specifically constructed for brain systems were used.

In summary, our study results demonstrated pattern topology alterations in the dopaminergic and cholinergic networks at different stages of dementia. MCI and AD could be associated with disease progression. The PHN analysis demonstrated the integration of subregions in the network into full connection at different scales and distinguished alterations in brain ^18^F-FDG PET metabolic connectivity as a model of dementia. We identified the dysregulation of the dopaminergic and cholinergic networks in the pathology of dementia.

## Methods

### Participants

We recruited 73 individuals and obtained ^18^FDG-PET data for them. The clinical evaluation performed in this study revealed that 16 individuals had early AD, 18 had MCI, 16 had SCD, and 23 were HCs. The detailed cohort information and Mini-Mental State Examination results^40^ are presented in Table 1. Age, sex, height, weight, and body mass index did not differ significantly among the four groups. An AD diagnosis was established during a multidisciplinary consensus meeting, according to the clinical criteria for probable AD from the National Institute on Aging–Alzheimer’s Association^41^. MCI was diagnosed according to the revised consensus criteria from 2004^42^. The criteria for SCD in pre-MCI were based on a previous study^43^. All participants or their caregivers provided written informed consent for the experimental procedures, which were approved by the ethics committee of the Taipei Veterans General Hospital. All methods were carried out in accordance with the relevant guidelines and regulations of this ethics committee. The protocol conformed to the ethical standards of the Declaration of Helsinki for the protection of human participants.

**Table 1:** Demographics and clinical characteristics of study participants.

Characteristic	Early AD	MCI	SCD	Controls	*P* value
	(n = 16)	(n = 18)	(n = 16)	(n = 23)	
Age (years)	74.3 ± 6.1	70.6 ± 6.9	70.1 ± 6.1	69.1 ± 5.3	0.551
Gender	6 M/10F	8 M/10F	4 M/12F	6 M/ 17F	0.077
Education (years)	11.0 ± 4.4	12.0 ± 3.9	11.9 ± 5.7	12.6 ± 4.0	0.715
Height (cm)	158.1 ± 10.1	160.4 ± 8.8	156.4 ± 8.8	157.4 ± 6.5	0.532
Weight (kg)	57.3 ± 13.9	65.3 ± 13.1	59.1 ± 8.4	59.9 ± 7.8	0.168
BMI	22.7 ± 3.9	25.3 ± 4.1	24.2 ± 3.2	24.3 ± 3.3	0.250
MMSE score	22.6 ± 2.0	26.9 ± 2.1	27.7 ± 1.8	28.4 ± 1.5	< 0.001*

*AD* Alzhimer's disease; *MCI* mild cognitive impairment; *SCD* subjective cognitive decline; *MMSE* mini-mental state examination.

*Significance was tested using Mann–Whitney *U* and chi-square tests of differences between groups.

### MR and ^18^FDG-PET data acquisition and preprocessing

Metabolic and structural images were obtained using a hybrid 3 T Signa PET/MR scanner (GE Healthcare, Milwaukee, WI, USA), which could simultaneously acquire both time-of-flight PET and high-resolution magnetic resonance (MR) data. All participants fasted for six hours before the ^18^F-FDG PET injection (~ 185 MBq), and all PET scans started approximately 60 min after the injection. T1-weighted images were obtained using the 3D BRAVO sequence (TR = 8 ms; TE = 3 ms; FA = 12°; FOV = 25.6; and 192 slices with a thickness of 1 mm). The T1-weighed structural images were processed using the CAT 12 toolbox^44^ in Matlab (MathWorks, Natick, MA, USA) and mapping to the Montreal Neurological Institute (MNI) template. The brain structural data were then segmented into areas of grey matter, white matter, and cerebrospinal fluid. Bias correction and modulation were performed to remove intensity nonuniformities in the grey matter. Finally, all the images were smoothed using a Gaussian filter (8 mm full width at half maximum [FWHM]).

The ^18^F-FDG PET images were co-registered with the T1‐weighted MR images and then spatially normalised to the MNI atlas using the software toolkit Statistical Parametric Mapping (SPM12, Wellcome Department of Cognitive Neurology, London, UK) in Matlab. The normalised ^18^F-FDG PET images were corrected for partial volume effects and the PETPVE12 toolbox was applied^45^. Subsequently, the standardised uptake values of the ^18^F-FDG PET images were normalised to the mean value for the cerebellum. We conducted spatial smoothing with a Gaussian kernel for the FWHM equal to (8,8,8) in three directions (x,y,z) to improve the signal-to-noise ratio.

### Node selection and network construction

We focused on the neurotransmitter networks related to dementia pathology and selected the cholinergic and dopaminergic neurotransmission pathways. We created each module of the metabolic connectivity network according to the method proposed by Caminiti et al.^46^.

**Figure 3:**
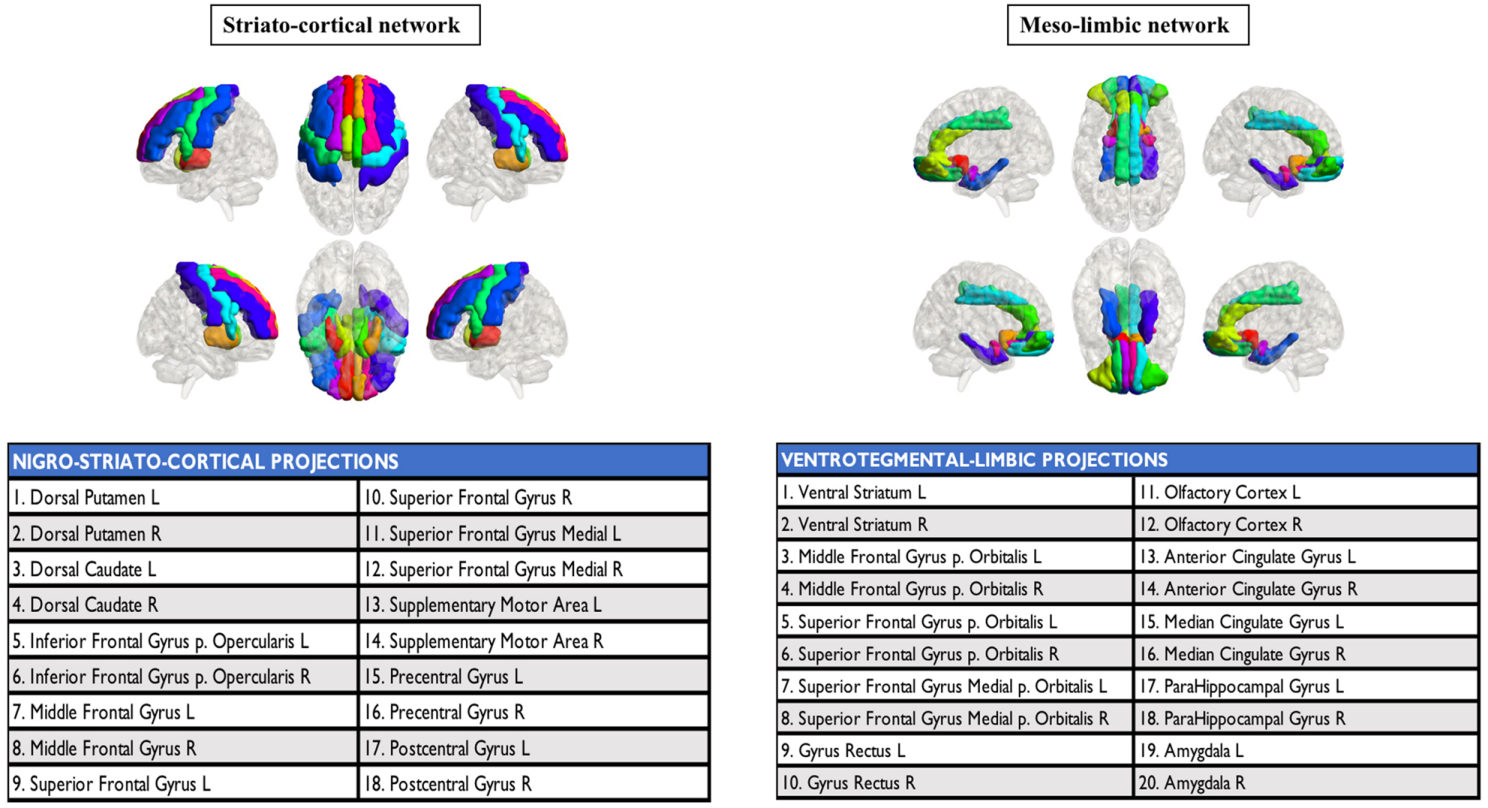
Dopaminergic networks. Cortical and subcortical projections of the two main dopaminergic pathways. R: right, L: left.

For the dopaminergic network analysis, we created striatocortical and mesolimbic networks (Fig. 3). The striatocortical network consisted of the dorsal caudate and dorsal putamen, frontal premotor, motor, executive dorsolateral frontal regions, and somatosensory cortex. The mesolimbic network consisted of the ventral striatum, ventral and medial frontal areas, anterior and middle cingulate cortices, amygdala, and parahippocampal cortex.

**Figure 4:**
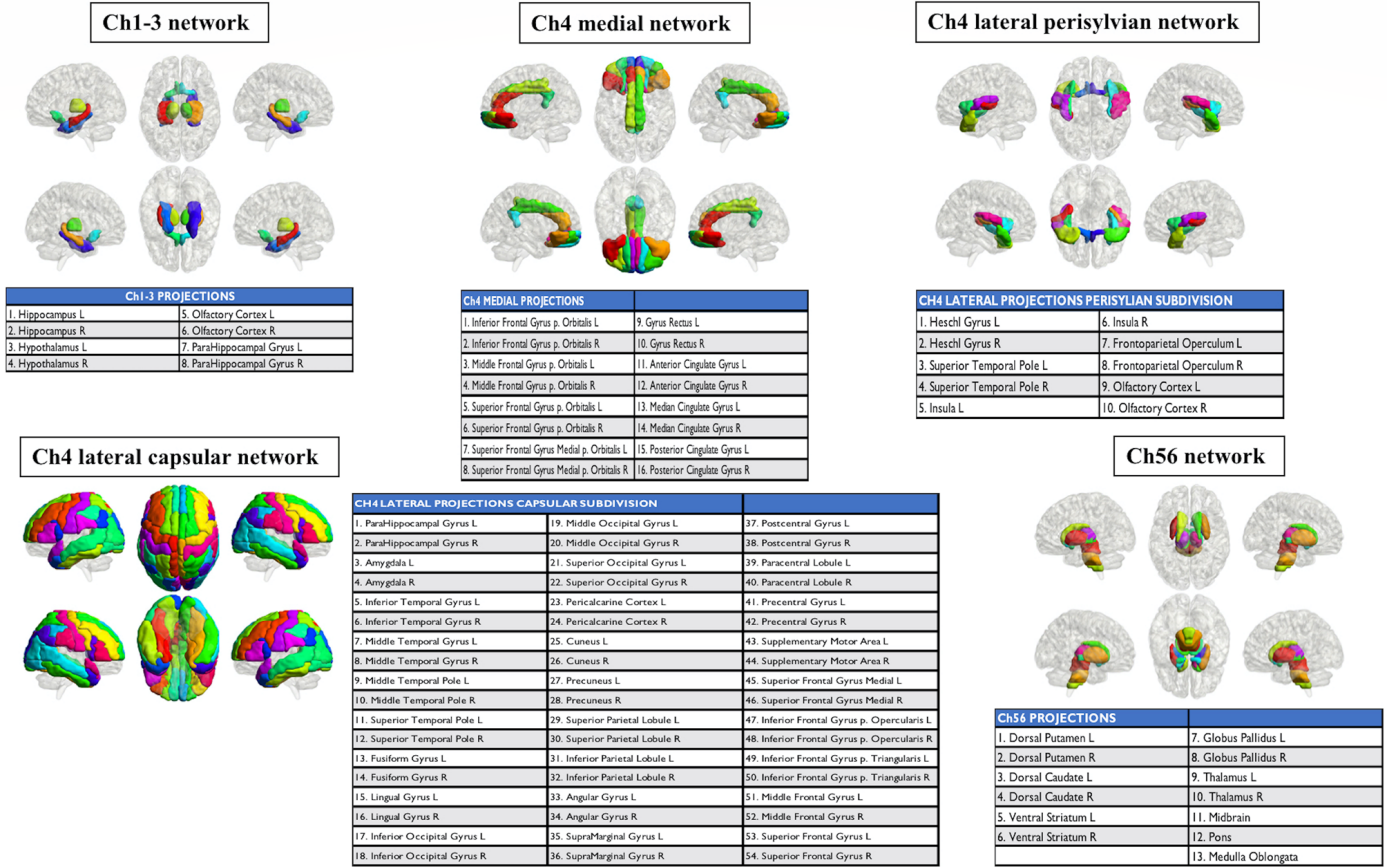
Cholinergic networks. Cortical and subcortical projections of the six main cholinergic pathways. Ch1–Ch2 and Ch3 are basal forebrain regions and Ch5–Ch6 are brainstem nuclei. Three pathways innervate the nucleus basalis of Meynert (Ch4). R: right, L: left.

For the cholinergic network analysis, we created five cholinergic networks (Fig. 4). The first network consisted of brain regions innervated by Ch1, including the Ch2 nuclei of the forebrain, namely the bilateral hippocampus and hypothalamus, and the pathway arising from the Ch3 nucleus, which includes the olfactory and parahippocampal cortices.

The cholinergic neurons in the brainstem pathways are the Ch5 pedunculopontine tegmental nucleus and the Ch6 laterodorsal tegmental grey of the periventricular area, which extend to the thalamus, ventral and dorsal striatum, globus pallidus, and the brainstem reticular formation, which is represented by the pons, midbrain, and medulla oblongata.

The other three cholinergic networks were represented by pathways originating from the Ch4 nuclei. The first Ch4 medial pathway reaches the cingulate, retrosplenial, and orbitofrontal cortices. The second Ch4 lateral perisylvian division joins the olfactory and superior temporal cortices with the insula and frontoparietal operculum. The third Ch4 lateral capsular division connects to the remaining frontal, parietal, temporal, and occipital cortices, as well as the amygdala.

We created dopaminergic and cholinergic subject-by-node/region-of-interest (ROI) matrices for each group. The matrices contained regional cerebral metabolic values derived from each subject in each node/ROI.

### PHN analysis

**Figure 5:**
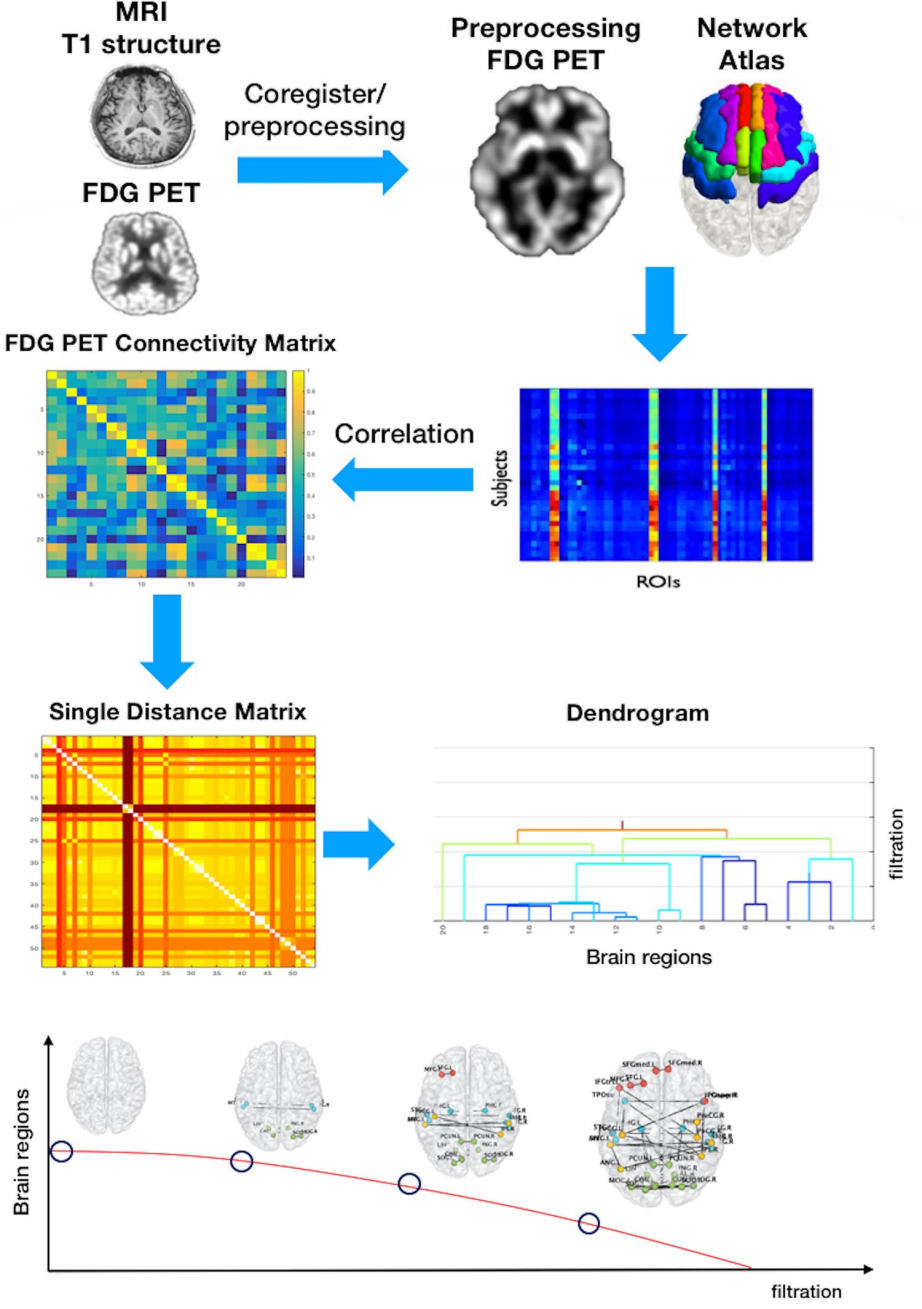
Flowchart of the persistent homology network (PHN) analysis methodology. Each group of preprocessed ^18^F-FDG PET images had regions of interest extracted within networks, calculated using Pearson’s correlation coefficients between intra-networks and across individuals. We converted the correlation-based matrix to a distance matrix using the formula 1—|correlation|. Using zeroth Betti numbers to create a persistent homology graph of filtration enabled us to track the construction of all regions connected to the network as the filtration value increased.

A flowchart summarising the PHN analysis methodology is presented in Fig. 5. Each group of preprocessed ^18^F-FDG PET images had ROIs extracted from the cholinergic and dopaminergic networks, and Pearson’s correlation coefficients between intra-networks were calculated across individuals. To calculate the PHN, the distances between regions were measured. We converted the correlation-based matrix to a distance matrix using the formula 1 −|correlation|. The PHN enabled us to quantify various persistent topological features based on graph filtration (λ). Using zeroth Betti numbers to create a persistent homology graph of filtration enabled us to track the construction of all regions connected to the network as the filtration value increased^47^. During this construction, we could record the start, the time at which a region appeared, and the end for various filtration values. With respect to filtration, when λ = 0, all regional components are loose: the number of connected components decreases with an increase in filtration. We can use the SIP as a rate of component convergence. However, the slope is not the most appropriate method to describe the plot, since it is nonlinear. We considered the AUC of the SIP to represent the sum of the distances of all paths. The AUC of the SIP is equal to efficient glucose metabolism demand within the network due to weak and strong connections.

Each topological feature was displayed using a bar that started and ended when the feature appeared and disappeared, respectively. This bar code was used to quantify and visualise evolutionary changes in topological features that occurred when the graph filtration changed. By incorporating additional geometric information into the bar code, we obtained an SLD and a dendrogram that showed the overall evolution and the hierarchical connections between the components of the network. We visualised these changes during filtration using the bar codes and dendrograms. The bar codes decreased with the threshold for ‘the number of regions’ to 1. If the network was strongly connected at a small threshold of distance (dij), the SIP decreased rapidly.

### Graph theory analysis

To identify the persistent homology in the cholinergic and dopaminergic networks, the network must be represented as a simplicial complex. A distance function in the underlying network corresponds to a filtration of the simplicial complex. Graph theory analysis has three other graph-theoretical parameters related to the distance function to estimate intra-network parameters: CPL, ND, and EC. These parameters were obtained using the Brain Connectivity Toolbox, which is an open-source Matlab code (http://www.brain-connectivity-toolbox.net). CPL is considered the average shortest distance between all regional pairs^13^. For example, a low CPL indicates a network with easy or quick data transfer. ND indicates how far the furthest regions of a network are from one another, based on paired path lengths. It provides an understanding of the network size. A high ND and low CPL would, therefore, be considered to indicate an efficient network. EC assigns increased importance to regions that are connected to other highly connected regions through the intra-network^48^.

### Statistical analysis

Data analyses were conducted in two steps: demographic and main analyses. The demographic analysis compared demographic variables with the clinical characteristics of the four groups of patients (AD, MCI, SCD and HC) to identify any potentially confounding relationships. The demographic analyses were performed using Mann–Whitney *U* and chi-square tests to identify differences among the groups. We used SPSS 13 software (SPSS, Chicago, IL, USA) for the analyses, with statistical significance assumed at *P* < 0.05^49^. To obtain stable results in the main analysis of the network parameters, we used a permutation test considering 10,000 permutations between any two groups in addition to significant differences between any pair of groups at a significance level of 0.001. This statistical method has been described in a previous study^50^.

## Supplementary Information

Supplementary Information
