# Supplementary material for: Disrupted metabolic connectivity in dopaminergic and cholinergic networks at different stages of dementia from 18F-FDG PET brain persistent homology network
Source: Sci Rep. 2021 Mar 8;11:5396. doi: 10.1038/s41598-021-84722-8 (PMC7940645; doi:10.1038/s41598-021-84722-8)

Disrupted Metabolic Connectivity in Dopaminergic and Cholinergic Networks at Different Stages of Dementia from ^{18}F-FDG PET Brain Persistent Homology Network

Tun-Wei Hsu[1,7], Jong-Ling Fuh [2,3,4], Da-Wei Wang[5], Li-Fen Chen[6], Chia-Jung Chang [7,8], Wen-Sheng Huang [7,8], Hsiu-Mei Wu[1,2], Wan-Yuo Guo[1,2]

[1]Department of Radiology, Taipei Veterans General Hospital, Taipei, Taiwan
[2]Faculty of Medicine, School of Medicine, National Yang-Ming University, Taipei, Taiwan
[3]Division of General Neurology, Neurological Institute, Taipei Veterans General Hospital, Taipei, Taiwan
[4]Brain Research Center, National Yang-Ming University, Taipei, Taiwan
[5]Institute of Information Science, Academia Sinica, Taipei, Taiwan
[6]Institute of Brain Science, School of Medicine, National Yang-Ming University, Taipei, Taiwan
[7]Department of Nuclear Medicine, Taipei Veterans General Hospital, Taipei, Taiwan
[8]Integrated PET/MR Imaging Center, Taipei Veterans General Hospital, Taipei, Taiwan

E-mail:
shaubow@gmail.com
stellafuh@gmail.com
wdw@iis.sinica.edu.tw
lfchen.tw@gmail.com
ohbabe0201@gmail.com
wshuang01@gmail.com
mei21030@gmail.com
wyguo@vghtpe.gov.tw

Jong-Ling Fuh and **Hsiu-Mei Wu** contributed equally to this work and share the role of corresponding authors.

Correspondence: Jong-Ling Fuh, MD, Ph.D.
Department of Neurology, Neurological Institute, Taipei Veterans General Hospital,
No.201, Sec. 2, Shipai Rd., Beitou District, Taipei, Taiwan 11217.
Tel: ; Fax: ;
E-mail: stellafuh@gmail.com

Correspondence: Hsiu-Mei Wu, MD.
Department of Radiology, Taipei Veterans General Hospital,
No.201, Sec. 2, Shipai Rd., Beitou District, Taipei, Taiwan 11217.
Tel: # ; Fax: ;
E-mail: mei21030@gmail.com

Study Funding:
The study was supported by grants from the Academia Sinica of Taiwan (AS-BD-108-2), the Ministry of Science and Technology of Taiwan (107-2221-E-075-006, 109-2314-B-075-052-MY2), the Taipei Veterans General Hospital (V108C-113, VGHUST109-V1-5-1, V109C-061), and the Brain Research Center, National Yang-Ming University from The Featured Areas Research Center Program within the framework of the Higher Education Sprout Project by the Ministry of Education (MOE) in Taiwan.

Table S1 The significance of each index of permutation for between-group differences in different networks . The black color indicate the significant pairs based on at least 10,000 permutations.

Networks and parapmeters	Group				Pairs of permutation test ($p < 0.001$)					
	**AD**	**MCI**	**SCD**	**HC**	AD vs HC	MCI vs HC	SCD vs HC	AD vs SCD	AD vs MCI	MCI vs SCD
**Dopaminergic network (striato-cortical)**										
Network characteristic path length	0.51	0.69	0.44	0.28		■				
Network diameter	1.26	1.36	1.15	0.75						
Eigenvector centrality	0.23	0.23	0.23	0.23						
SIP AUC	3.01	4.33	2.42	1.72		■				■
**Dopaminergic network (meso-limbic)**										
Network characteristic path length	0.53	0.74	0.49	0.56						■
Network diameter	1.11	1.53	1.13	1.31						
Eigenvector centrality	0.22	0.22	0.22	0.22						
SIP AUC	2.23	2.91	1.56	1.69						
**Cholinergic network (Ch1-3 pathway)**										
Network characteristic path length	0.48	0.43	0.30	0.32						
Network diameter	1.07	1.02	0.80	0.80						
Eigenvector centrality	0.35	0.35	0.34	0.34						
SIP AUC	1.14	1.10	0.55	0.63				■		
**Cholinergic network (Ch4 medial pathway)**										
Network characteristic path length	0.57	0.71	0.48	0.57				■		■
Network diameter	1.30	1.48	1.13	1.17						
Eigenvector centrality	0.25	0.25	0.25	0.25						
SIP AUC	1.72	2.19	1.52	1.38		■				
**Cholinergic network (Ch4 lateral perisylvian)**										
Network characteristic path length	0.41	0.31	0.28	0.29						
Network diameter	0.70	0.55	0.66	0.63						
Eigenvector centrality	0.31	0.31	0.31	0.31						
SIP AUC	1.46	1.33	0.93	0.91						
**Cholinergic network (Ch4 lateral capsular)**										
Network characteristic path length	0.54	0.63	0.58	0.43						
Network diameter	1.24	1.52	1.35	1.28						
Eigenvector centrality	0.13	0.13	0.13	0.13						
SIP AUC	8.04	10.64	6.84	5.71		■				■
**Cholinergic network (Ch5-6 pathway)**										
Network characteristic path length	0.42	0.31	0.26	0.29						
Network diameter	1.16	0.67	0.70	1.22						
Eigenvector centrality	0.27	0.27	0.27	0.26						
SIP AUC	1.75	1.53	1.05	1.37				■		

Figure S1. Dopaminergic network analysis. The mesolimbic network was affected in the MCI group, showing a delay in connectivity and a change in configuration in the frontal lobe, amygdala, and anterior and posterior cingulate cortex. (**a**) Single linkage distance (SLD) in the Alzheimer's disease (AD), mild cognitive impairment (MCI), subjective cognitive decline (SCD), and healthy control (HC) groups, obtained from the original correlation-based distance. (**b**) Single linkage dendrograms of the AD, MCI, SCD, and HC groups. The vertical and horizontal axes represent the brain region index (as in Fig. 3) and graph filtration. The connected network is divided into smaller regions when the distance (graph filtration) decreases. (**c**) The corresponding persistent features of each group at different scales of graph filtration and (**d**) mesolimbic network maps constructed using five different filtration values: 0.05, 0.10, 0.15, 0.20, and 0.25.

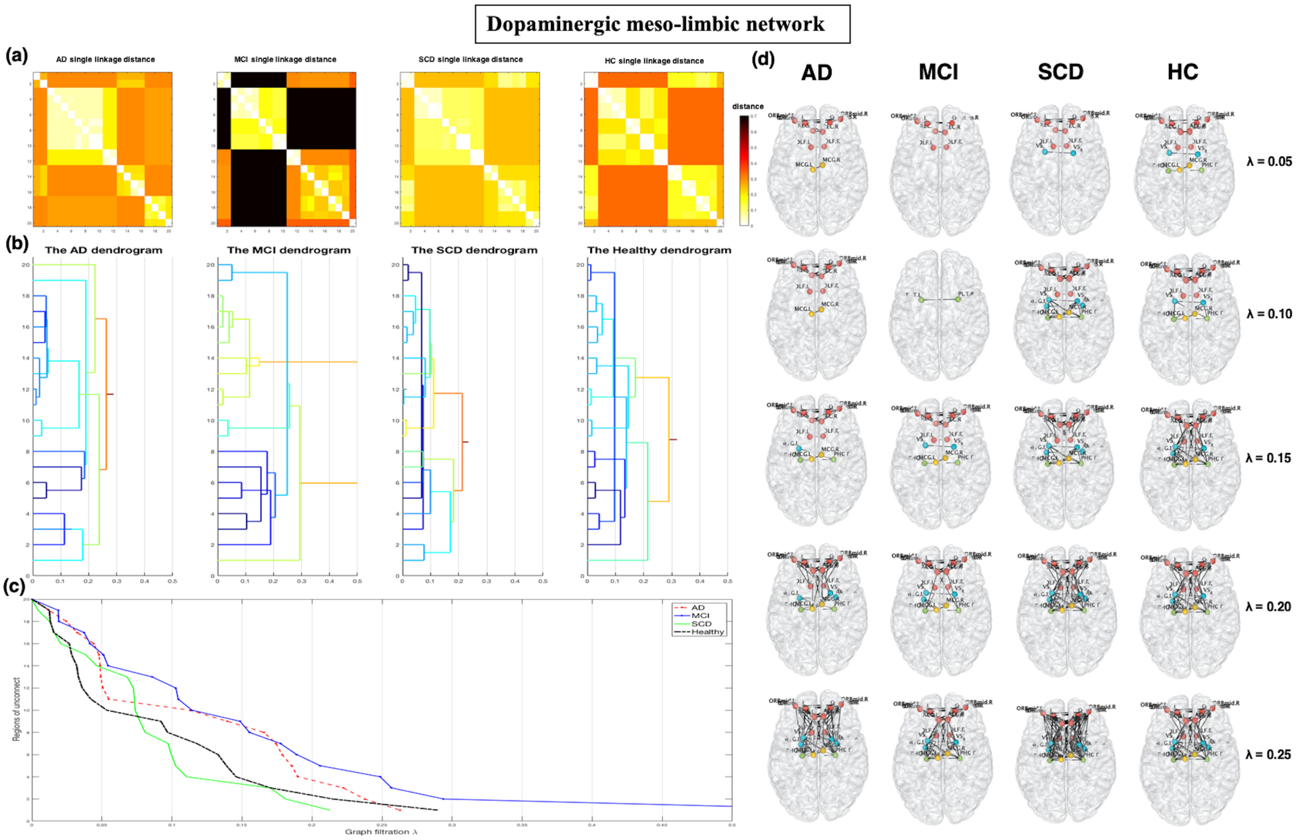

Figure S2. Cholinergic network analysis shows that the pathways of the Ch1–2 and Ch3 basal forebrain were affected in the Alzheimer's disease (AD) and mild cognitive impairment (MCI) groups, indicating that clustering occurred later in the hippocampus and para-hippocampus. (a) Single linkage distance (SLD) in the AD, MCI, subjective cognitive decline (SCD) and healthy control (HC) groups, obtained from the original correlation-based distance. (b) Single linkage dendrograms for the AD, MCI, SCD and HC groups. The vertical and horizontal axes represent brain region index (as in Fig. 4) and graph filtration. The connected network is divided into smaller regions when the distance (graph filtration) decreases. (c) The corresponding persistent features of each group at different scales of graph filtration and (d) Ch1–3 network maps constructed using five different filtration values: 0.05, 0.10, 0.15, 0.20, and 0.25.

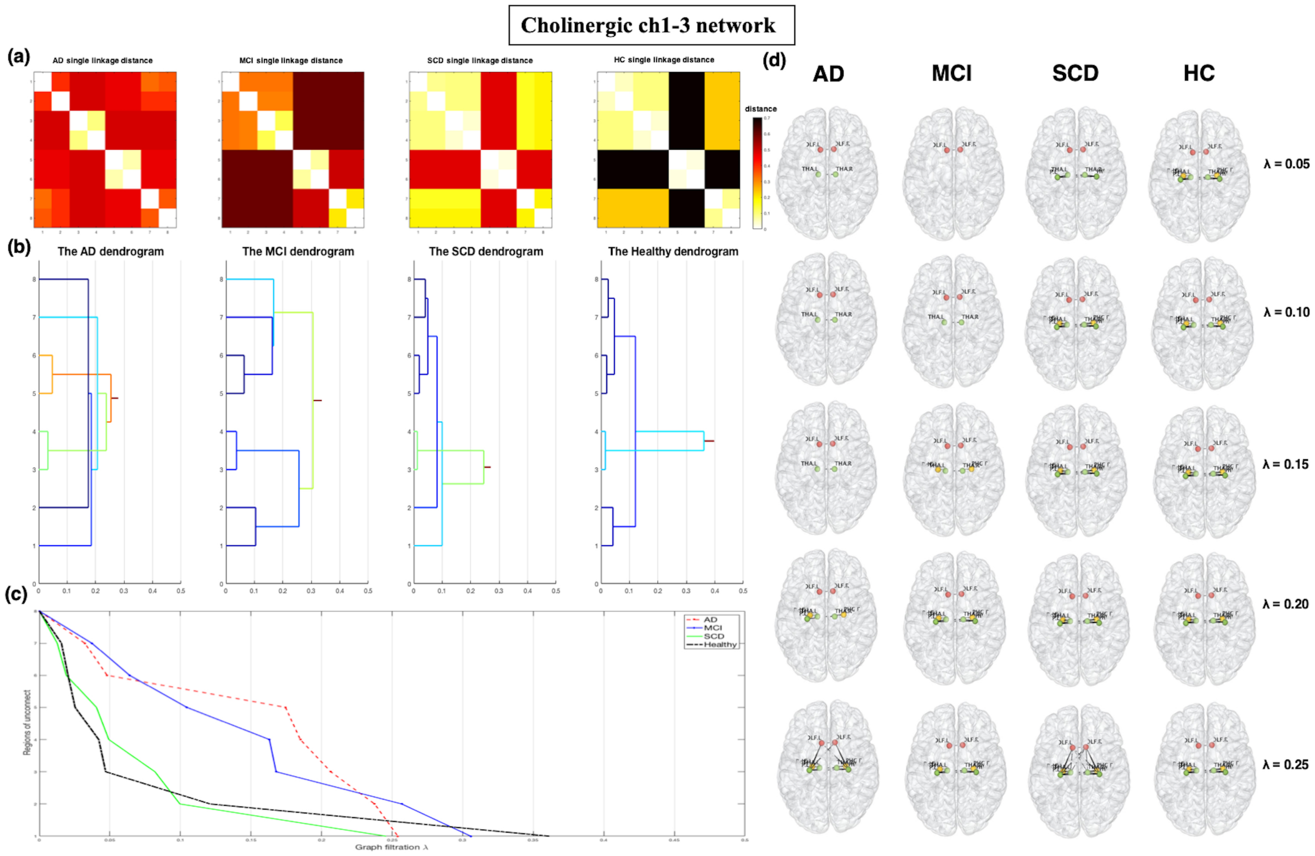

Figure S3. Cholinergic network analysis shows that the pathways of the Ch4 medial projection were affected in the Alzheimer's disease (AD) and mild cognitive impairment (MCI) groups, indicating that clustering occurred later in the frontal gyrus. (a) Single linkage distance (SLD) in the AD, MCI, subjective cognitive decline (SCD) and healthy control (HC) groups, obtained from the original correlation-based distance. (b) Single linkage dendrograms for the AD, MCI, SCD and HC groups. The vertical and horizontal axes represent brain region index (as in Fig. 4) and graph filtration. The connected network is divided into smaller regions when the distance (graph filtration) decreases. (c) The corresponding persistent features of each group at different scales of graph filtration and (d) Ch4 medial projection network maps constructed using five different filtration values: 0.05, 0.10, 0.15, 0.20, and 0.25.

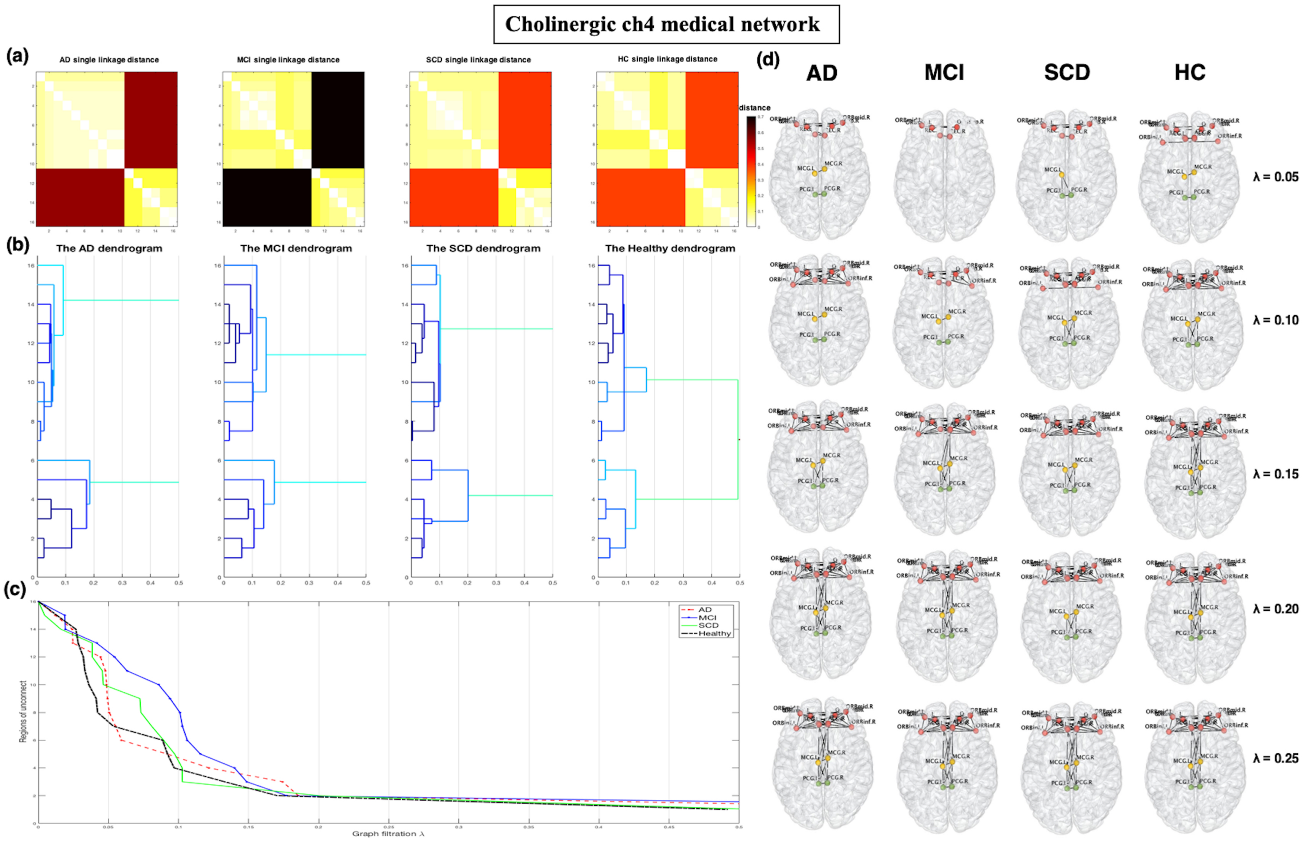

Figure S4. Cholinergic network analysis shows that the pathways of the Ch4 lateral perisylvian division were affected in the Alzheimer's disease (AD) and mild cognitive impairment (MCI) groups, indicating longer distances in the Herschel gyrus, insula, and olfactory region. (a) Single linkage distance (SLD) in the AD, MCI, subjective cognitive decline (SCD) and healthy control (HC) groups, obtained from the original correlation-based distance. (b) Single linkage dendrograms for the AD, MCI, SCD and HC groups. The vertical and horizontal axes represent the brain region index (as in Fig. 4) and graph filtration. When the distance (graph filtration) decreases, the connected network is divided into smaller regions. (c) The corresponding persistent features of each group at different scales of graph filtration and (d) Ch4 lateral perisylvian projection network maps constructed using five different filtration values: 0.05, 0.10, 0.15, 0.20, and 0.25.

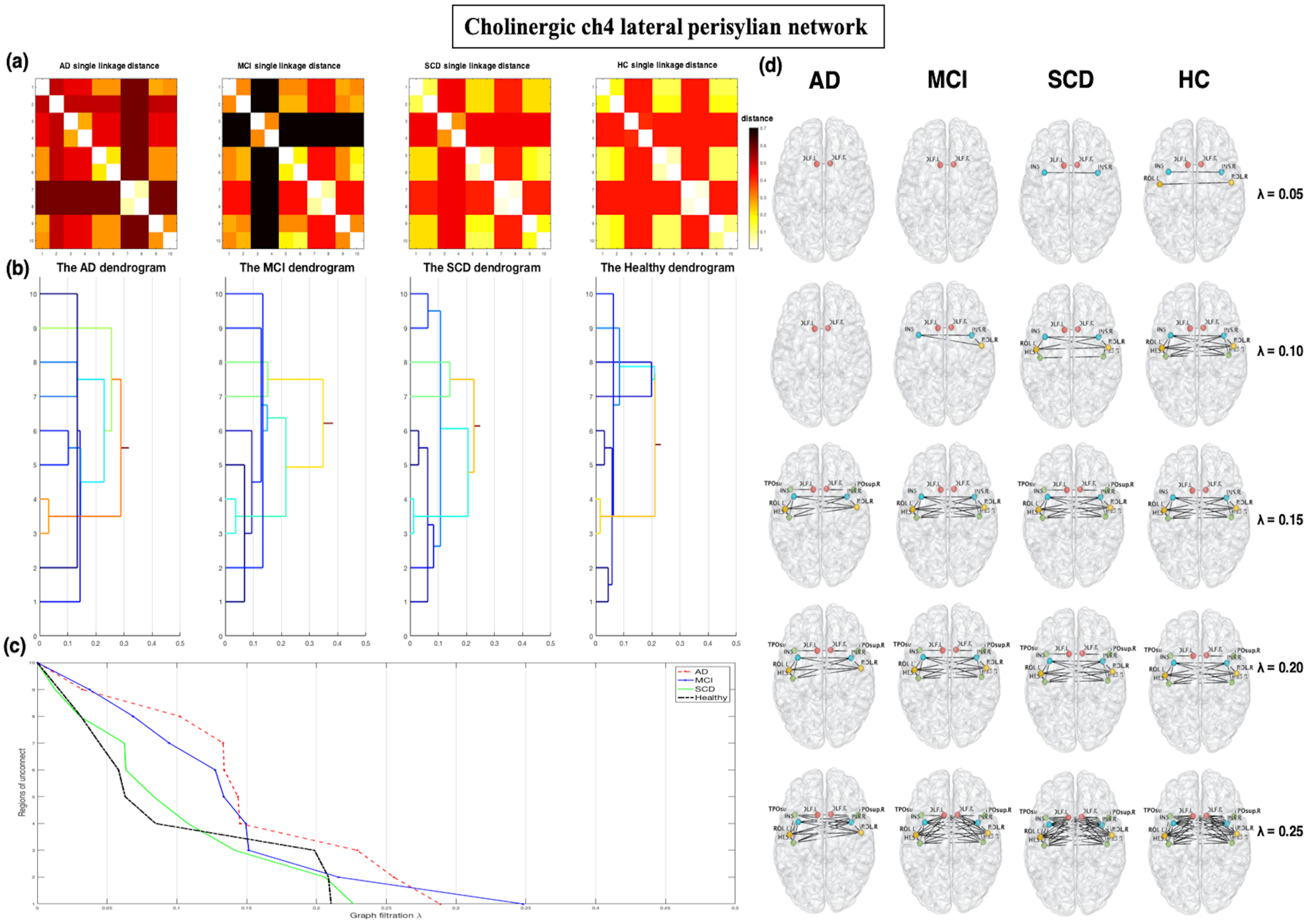

Figure S5. Cholinergic network analysis. The pathways of the Ch5–Ch6 brainstem nuclei reveal longer distances in the ventral striatum and brainstem in the Alzheimer's disease (AD) and mild cognitive impairment (MCI) groups. (a) Single linkage distance (SLD) in the AD, MCI, subjective cognitive decline (SCD) and healthy control (HC) groups, obtained from the original correlation-based distance. (b) Single linkage dendrograms for the AD, MCI, SCD and HC groups. The vertical and horizontal axes represent the brain region index (as in Fig. 4) and graph filtration. When the distance (graph filtration) decreases, the connected network is divided into smaller regions. (c) The corresponding persistent features of each group at different scales of graph filtration and (d) Ch5–Ch6 network maps constructed using five different filtration values: 0.05, 0.10, 0.15, 0.20, and 0.25.

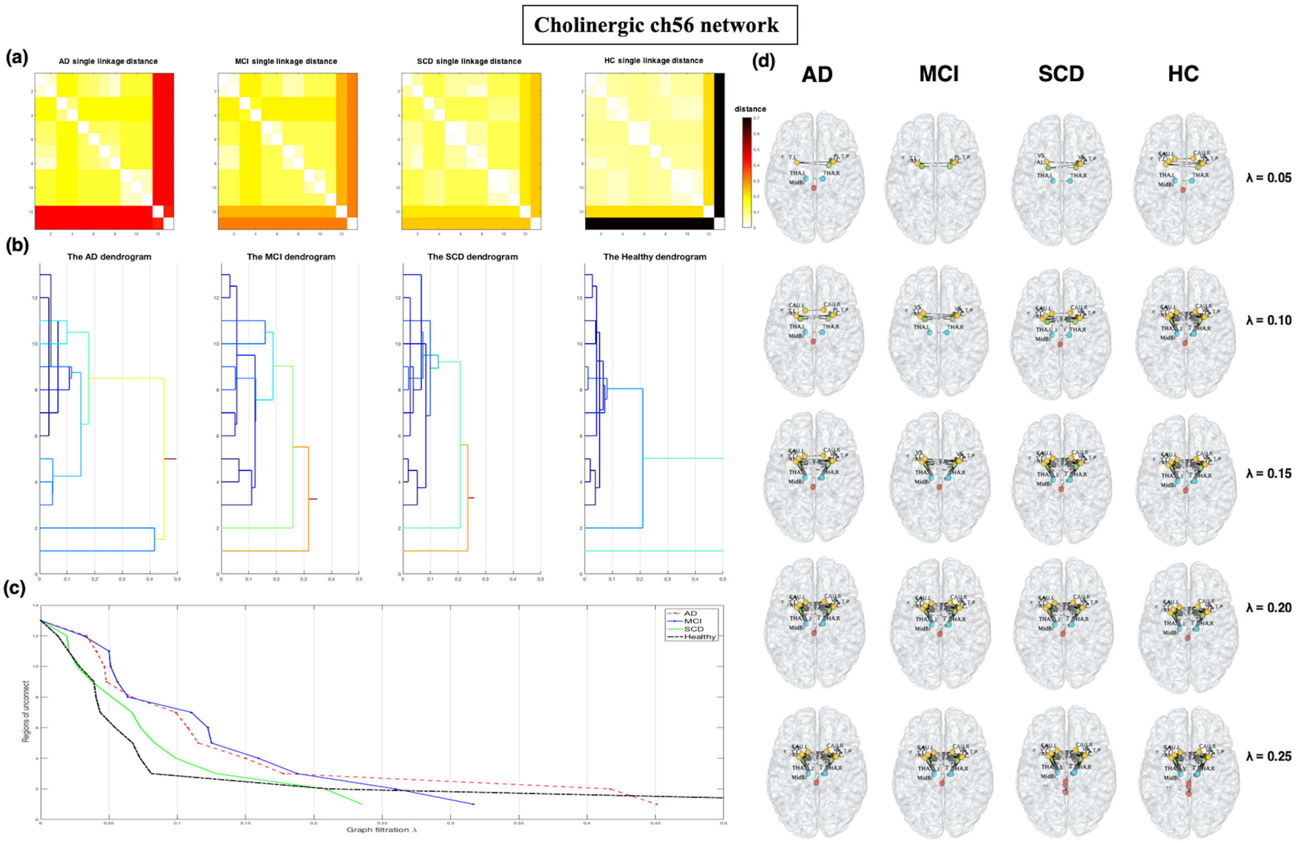

Supplement: Supplementary file 1 — Supplementary Information [file 41598_2021_84722_MOESM1_ESM.pdf]
